# Capacitive Saccharide Sensor Based on Immobilized Phenylboronic Acid with Diol Specificity

**DOI:** 10.1007/s12010-018-2911-3

**Published:** 2018-10-28

**Authors:** Gizem Ertürk Bergdahl, Martin Hedström, Bo Mattiasson

**Affiliations:** 1 CapSenze Biosystems AB, Scheelevägen 22, 22363 Lund, Sweden; 2 Department of Biotechnology, Kemicentum, Lund University, Sölvegatan 39A, 22100 Lund, Sweden; 3 Department of Clinical Sciences, Lund University, Tornavägen 10, 22184 Lund, Sweden

**Keywords:** Capacitive sensor, Saccharide detection, Aminophenylboronic acid, Glycoprotein detection

## Abstract

A capacitive sensor for saccharide detection is described in this study. The detection is based on selective interaction between diols and aminophenylboronic acid (APBA) immobilized on a gold electrode. Glucose, fructose, and dextran (MW: 40 kDa) were tested with the system over wide concentration ranges (1.0 x 10^−8^ M - 1.0 x 10^−3^ M for glucose, 1.0 x 10^−8^ M - 1.0 x 10^−2^ M for fructose and 1.0 x 10^−10^ M - 1.0 x 10^−5^ M for dextran). The limits of detection (LODs) were 0.8 nM for glucose, 0.6 nM for fructose, and 13 pM for dextran. These data were comparable to the others reported previously. In order to demonstrate glycoprotein detection with the same sensor, human immunoglobulin G (IgG) as well as horseradish peroxidase were used as model analytes. The sensor responded to IgG in the concentration range of 1.0 x 10^−13^ M - 1.0 x 10^−7^ M with a LOD value of 16 fM. The performance of the assay of peroxidase was compared to a spectrophotometric assay by determining the enzymatic activity of a captured analyte. The results showed that the method might be useful for label-free, fast, and sensitive detection of saccharides as well as glycoproteins over a wide concentration range.

**Electronic supplementary material:**

The online version of this article (10.1007/s12010-018-2911-3) contains supplementary material, which is available to authorized users.

## Introduction

Saccharides are some of the most important biomolecules which play a fundamental role in biological systems [1] such as cell-to-cell interactions and biological recognition [2, 3]. Moreover, since a range of compounds are glycosylated and in some cases the level of glycosylation gives valuable clinical information, measurement of glycosylation level is very important in biological systems. A very clear example is to monitor the level of exposure to high glucose concentrations over time, and measurement of glycated hemoglobin (HbA1c) which gives an integrated measure over exposure during a period of at least 3 months [4–6]. It ought to be possible to register the presence of this kind of glycated compounds with sensitive and convenient assays.

Biological recognition elements especially lectins have been widely used in saccharide sensors for saccharide detection. However, boronic acid–based synthetic recognition elements are better choices with regard to stability and cost [7]. All these advantages showed promising results for saccharide detection. The reversible covalent interaction of boronic acids with cis-1,2- or 1,3-diols forms very strong binding affinity for saccharides in mM or sub-mM levels. Therefore, an increasing interest has been seen for the use of boronic acid and its derivatives as recognition elements for saccharide detection. Applications are seen in affinity chromatography, selective aggregation of polymers, in sensors, etc. [8–15]. Boronic acid chemistry has been employed in various sensing strategies including colorimetry [16], fluorimetry [17–21], surface plasmon resonance (SPR) [22–24], quartz crystal microbalance (QCM) [22], and electrochemistry [25–27].

The use of electrochemical techniques in saccharide detection has several advantages over other methods such as being simple, rapid, sensitive, and low-cost analysis [28–30]. Aytaç et al. reported a potentiometric saccharide sensor based on N-phenylboronic acid substituted polypyrrole for saccharide detection. They reported the limit of detection (LOD) values as 0.17 × 10^−3^ M and 0.008 × 10^−3^ M for d-fructose and d-glucose, respectively [25]. Pablos et al. reported a solid sensory kit based on phenylboronic acid for the detection and quantification of glucose, fructose, and dopamine. LOD value was reported as 3–4 × 10^−4^ M for three of the analytes [15]. Assemblies of the 5-amino-2-fluorophenylboronic acid–modified silver nanoparticles were synthesized by Cao et al. for colorimetric sensing of glucose over a concentration range of 0–20 × 10^−3^ M at physiological pH of 7.4 and the LOD value was reported as 89.0 × 10^−6^ M [31].

In the present work, the aim was to develop a sensitive, label-free, and fast detection system for saccharide detection. For this purpose, capacitive sensors were used as the detection technology owing to their advantages including highly sensitive [32], easy and label-free detection, low-cost, and reduced sample volume [33–36].

## Materials and Methods

### Materials

3-Aminophenylboronic acid monohydrate (APBA), *N*-hydroxysuccinimide sodium salt (NHS), 1-(3-dimethylaminopropyl)-3-ethylcarbodiimide hydrochloride (EDC), d-glucose, d-fructose, Dextran from *Leuconostoc* spp. (Dextran 40, Mw ~ 40.000), sodium carboxymethyl cellulose (Na-CMC) (Mw ~ 90.000), and tyramine (99%, HOC_6_H_4_CH_2_CH_2_NH_2_) were obtained from Sigma-Aldrich (Steinheim, Germany). 1-Dodecanethiol was purchased from Aldrich (Deisenhofen, Germany). Human gamma globulin (human IgG) was purchased from Octapharma AB (Stockholm, Sweden). Peroxidase (POD) from horseradish was purchased from Sigma-Aldrich (Deisenhofen, Germany).

### Aminophenylboronic Acid (APBA) Modification of Capacitive Gold Electrodes

In the first step, gold electrodes were cleaned with various solutions for 10 min in each step in ultrasonic cleaner as described previously [37]. Following plasma cleaning of the electrodes (Mod. PDC-3XG, Harrick, NY), electro-polymerization of tyramine was performed as described in previous reports [34, 37, 38]. By this way, free primary amino groups were introduced on the surface via the deposition of poly-tyramine.

Then, sodium carboxymethyl cellulose (Na-CMC) was dissolved in 0.05 M sodium phosphate buffer (pH: 6.0) to a final concentration of 1.0% (*w*/*v*). Poly-tyramine coated electrodes were immersed in this solution for 60 min at room temperature. CMC is a derivative of cellulose formed by the introduction of carboxymethyl groups throughout the polymer backbone. By this way, carboxyl groups were introduced on the surface of the electrode. This treatment was parallel to what has been implemented in SPR where the sensor surface has been modified in a similar way [39]. In the next step, for the activation of carboxyl groups, electrodes were immersed in 1 mL of 0.05 M 1-(3-dimethylaminopropyl)-3-ethylcarbodiimide hydrochloride (EDC) and 1.0 mL of 0.03 M *N*-hydroxysuccinimide sodium salt (NHS) in phosphate buffer (pH: 6.0) for 2 h. NHS-activated carboxylic groups were then allowed to bind with the primary amino groups of APBA (40 mM) in phosphate buffer (10 mM, pH: 7.0) overnight, at room temperature. By the deprotonation of the activated carboxyl groups after APBA treatment, tetrahedral boronate anion, which interacts with the monosaccharides to form boronate-hydroxyl complexes, was introduced on the surface of the electrode. Finally, the APBA–modified electrode was treated with 1-dodecanethiol (10 mM) in ethanol for 20 min in order to ensure proper insulation of the gold electrodes.

**Scheme 1:**
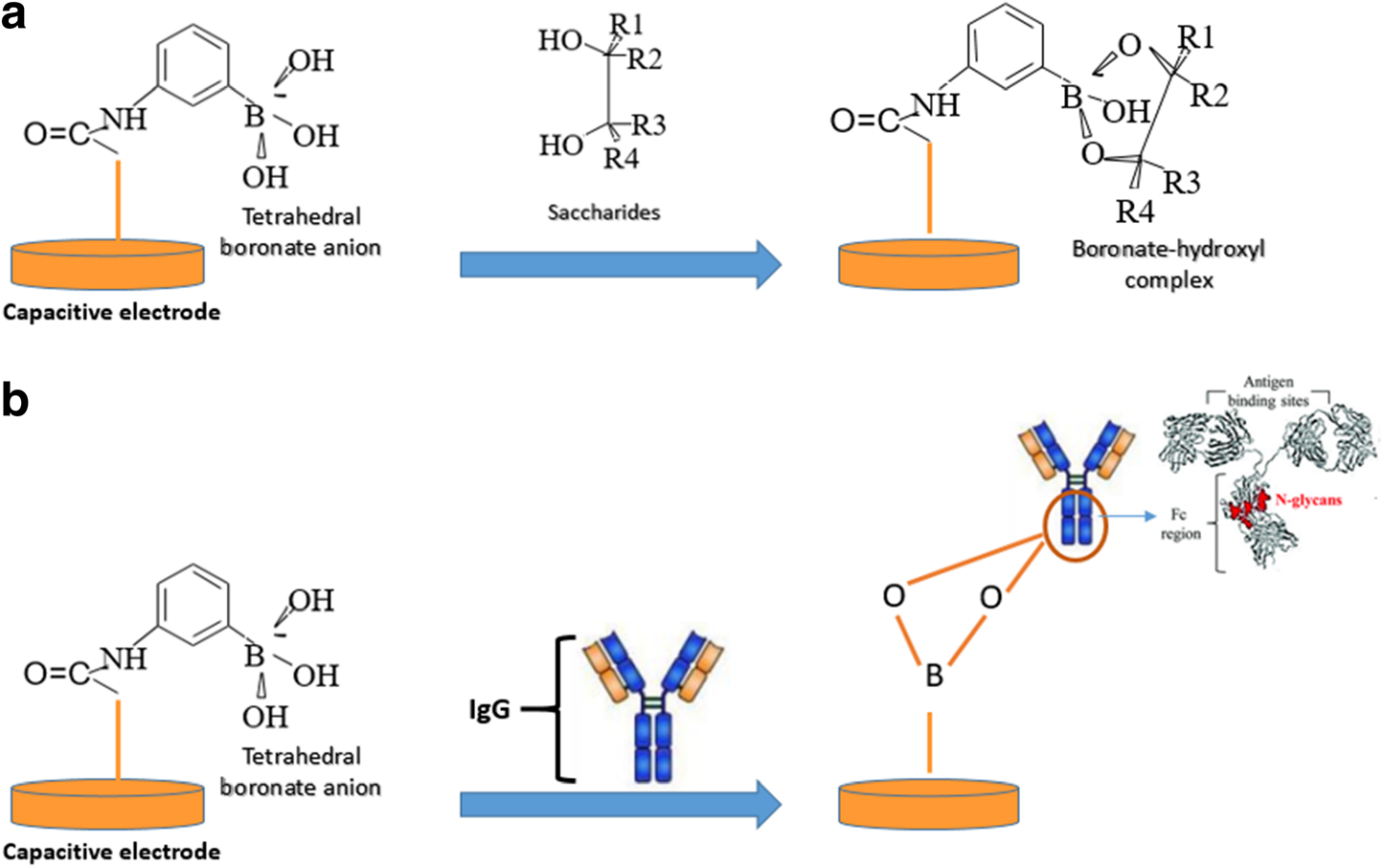
**A** Tetrahedral boronate anion formation on the capacitive gold electrode after APBA modification and interaction of them with **A** saccharides and **B** IgG

Formation of tetrahedral boronate anions on the capacitive gold electrode after APBA modification and interaction of these groups with saccharides and glycoproteins are shown schematically in Scheme 1.

### Characterization of APBA–Modified Electrodes

#### Cyclic Voltammetry (CV) Studies

Cyclic voltammetry (CV) based on potentiostat/galvanostat (Autolab PGSTAT 12, Ecochemie, Utrecht, Netherlands) was used for characterization of different immobilized layers and evaluation of the extent of insulation of the modified surface after each step. A platinum wire and a commercial Ag/AgCl electrode were used as the counter and reference electrodes, respectively for CV measurements as described in previous reports [40, 41]. A solution of KCl (0.1 M) containing 0.1 M (K_3_[Fe(CN)_6_]) was used as the electrolyte solution as has been reported before [38, 42].

#### Atomic Force Microscopy (AFM) Analysis

In order to register the change in surface morphology of bare gold electrode and APBA-modified electrode, atomic force microscope (AFM) (Veeco Instruments Inc., USA) in tapping mode was used. The scanning area was 10 μm × 10 μm and the vertical examination space was 2.5 μm in the analysis.

#### Scanning Electron Microscopy (SEM) Analysis

In order to characterize the surface morphology of bare electrode and APBA–modified electrode, SEM analyses were performed with Quanta 400 F Field Emission SEM (USA). For scanning electron microscopy, the electrodes were sputtered with gold/palladium.

### Capacitive Measurements with APBA–Modified Electrode

Capacitive measurements (CapSenze Biosystems, AB, Lund, Sweden) were performed with APBA–modified electrode inserted into the flow cell. Current pulse method was used for the measurements [43].

#### Real Time Saccharide Detection with APBA–Modified Electrode

The first step in sample analysis sequence was regeneration of the surface (10 mM phosphate, pH: 6.0) for 2.5 min. After a stable baseline was established by injection of running buffer (10 mM phosphate, pH: 10.0), standard solutions of different concentrations of glucose (1.0 × 10^−8^ M–1.0 × 10^−3^ M), fructose (1.0 × 10^−8^ M–1.0 × 10^−2^ M), and dextran (mw 40.000 D) (1.0 × 10^−10^ M–1.0 × 10^−5^ M) were injected into the system. When the target analyte was captured by the electrode, this resulted in a decrease in the registered capacitance of the system. This change was calculated as a function of time from the sensorgrams.

#### Real-Time Glycoprotein Detection with APBA–Modified Electrode

In order to demonstrate glycoprotein detection with the capacitive sensor, human immunoglobulin G (IgG) was selected as a model glycoprotein. For IgG detection, all of the experimental parameters were same with those used for the saccharide detection. IgG was analyzed as the standard solutions with the concentrations between 1.0 × 10^−13^ M and 1.0 × 10^−7^ M.

#### Comparison of Performance of APBA–Modified Capacitive System with Spectrophotometric Assay

First of all, APBA–modified electrodes were exposed to a pulse of HRP and the change in capacitance was registered. Then, the electrode was taken out for further analysis of HRP which was captured by the electrode. The HRP activity was measured by incubating the capacitive electrode with bound HRP in the substrates (TMB and hydrogen peroxide) [44]. First: 250 μl of a solution containing 1 mg·mL^−1^ of HRP was injected into the APBA–modified capacitive system and the capacitive change was registered. The amount captured was evaluated based on a calibration curve of signal amplitude vs concentration of HRP. Then, the electrode was removed without washing and incubated in 3.0 mL of substrate solution of TMB (0.416 μM) and hydrogen peroxide (0.832 μM) in phosphate buffer (10 mM, pH 7.0) at room temperature (25 °C). The activity was measured by registering at 650 nm. One enzyme unit was defined as the amount of enzyme converting 1 μmol of TMB per minute at 25 °C and pH 7.0.

## Results and Discussion

In the first step, the electrodes were coated with poly-tyramine layer. Then, carboxyl groups were introduced on the poly-tyramine layer by immobilizing carboxymethyl cellulose (CMS) and the carboxyl groups were activated by treatment with a mixture of 1-(3-dimethylaminopropyl)-3-ethylcarbodiimide hydrochloride (EDC) and *N*-hydroxysuccinimide (NHS) activation procedure. In the last step, aminophenylboronic acid (APBA) was immobilized on the NHS-activated surface. After detailed characterization of the APBA–modified electrodes, saccharide detection was performed from aqueous solutions of glucose, fructose, and dextran. Since the boronate functionalized sensor platform can interact with sugar ligands through covalent binding on the 1,2-cis-diol of glycol structure, immunoglobulin G (IgG) was chosen as a model glycoprotein to investigate the glycoprotein detection with the developed system. In the last step, performance of APBA–modified capacitive system was compared with the spectrophotometric method by comparing the amount of the captured glycoprotein, HRP, which is enzymatically active, to the registered enzymatic activity of the captured enzyme.

### Surface Characterization of APBA–Modified Electrodes

Surface characterization of APBA–modified electrodes was performed by using the methods listed below.

**Fig. 1:**
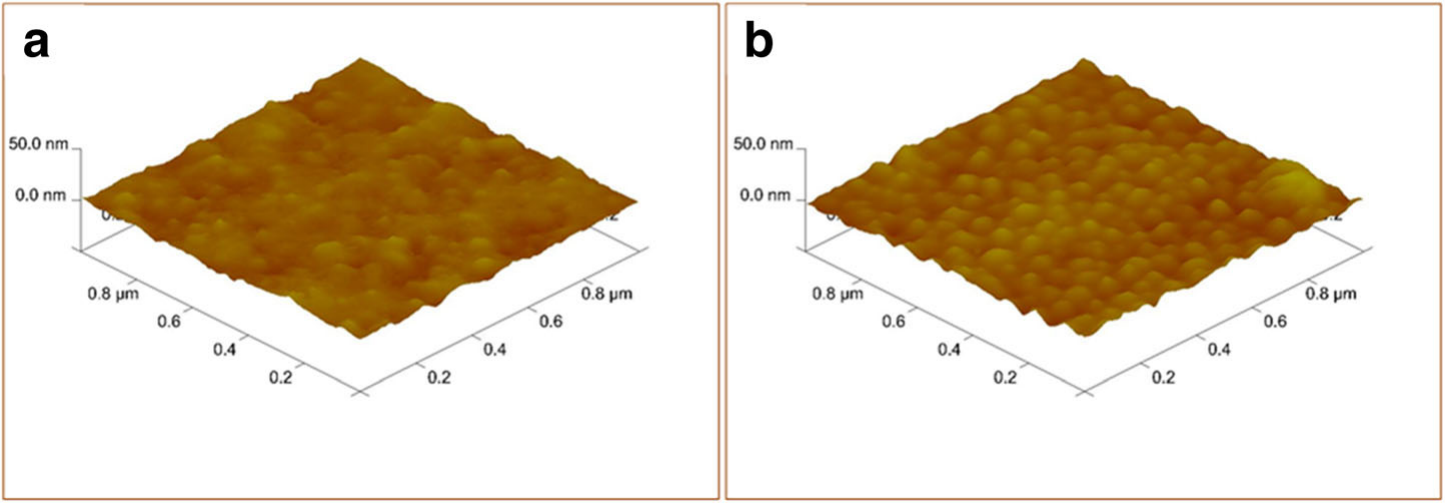
AFM images of unmodified (**A**) and APBA–modified electrode (**B**)

Proper insulation of the electrode surface is crucial in capacitive measurements [45, 46]. The degree of insulation of the electrodes was investigated by cyclic voltammetry (CV) using Fe(CN)_6_^4−/3−^ as the permeable redox couple. The scan range was between − 0.3 and + 0.8 V and the scan rate was 0.1 V·s^−1^. The results are shown in Supplementary Information (S.I. Fig. 1).

AFM images of unmodified gold electrode (Fig. 1A) and APBA–modified electrode (Fig. 1B) are shown in Fig. 1. The surface morphology and the surface topography changed after modification with APBA. It can be clearly seen from the images that a rough surface was obtained on the electrode after APBA modification. Detailed AFM images showing the phase and height are shown in Fig. SI.2 (Supplementary Information).

Figure SI.3 shows the SEM images of APBA–modified electrode at different magnifications. The rough surface can be clearly seen in the images and it indicates the successful modification of the surface with CMC+APBA.

### Real-Time Saccharide Detection with APBA–Modified Capacitive Sensor

In our experiments, current pulse method which was introduced by Erlandsson et al. [43] was used for capacitance measurements [47] .

In these systems, the capacitance is measured according to Eq. (1):

1 $$ \frac{1}{C(tot)}=\frac{1\ }{C\ (ins)}+\frac{1}{C\ (bio)}+\frac{1}{C\ (dl)} $$

where total change in capacitance is equal to the sum of capacitance contributions from different layers; (*C*_*ins*_), (*C*_*bio*_), and (*C*_*dl*_), respectively. According to this equation, when a target analyte binds to the surface, it will create a decrease in the total capacitance of the system via the displacement of the counter ions (diffuse layer) around the gold electrode [43].

**Fig. 2:**
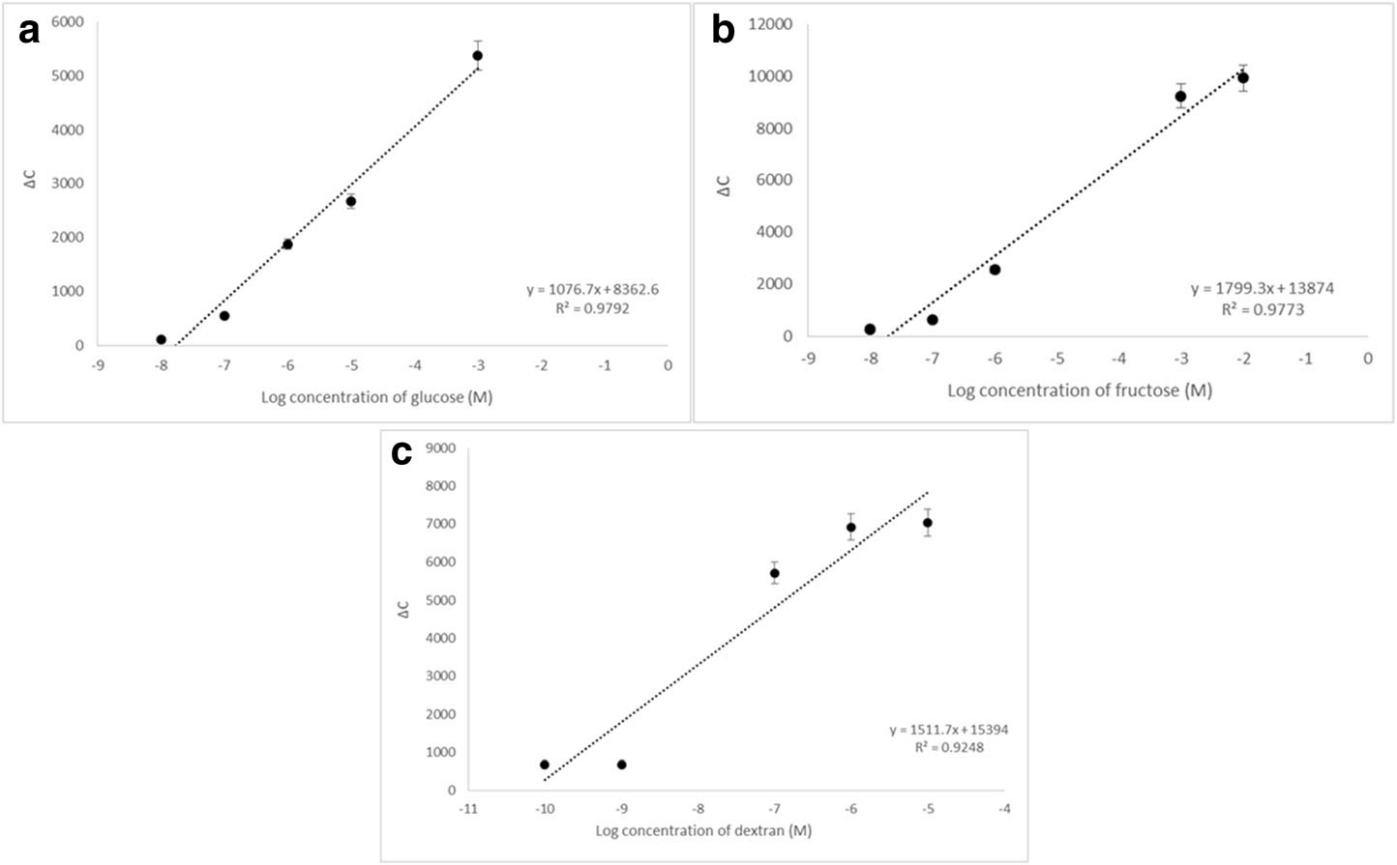
Capacitance change vs. logarithm of **A** glucose, **B** fructose, and **C** dextran concentrations for APBA–modified electrode under optimum conditions (flow rate, 100 μL·min^−1^; sample volume, 250 μL; running buffer, 10 mM phosphate; pH, 10.0; regeneration buffer, 10 mM phosphate; pH, 6.0; T, 25 °C)

In the principle of recognition in the capacitive measurements, boronic functional groups play a key role by forming complexes with compounds containing accessible diols through reversible ester formation. The resulting boronate anion is stable between pH range 6 and 10. It was observed that the affinity increased at higher pH values. This higher affinity to saccharides is due to the tetrahedral form of boronic acids in basic medium that shows high affinity to saccharides. In basic conditions, planar structure of boronic acid transforms to tetrahedral form and in this form, boronic acid covalently binds to cis-diols of saccharides. In the light of information reported in the previous studies [25, 26, 48], 10 mM phosphate buffer at pH: 10.0 was selected as the running buffer. Standard saccharide (glucose/fructose/dextran) solutions containing different concentrations of glucose (1.0 × 10^−8^ M–1.0 × 10^−3^ M), fructose (1.0 × 10^−8^ M–1.0 × 10^−2^ M), and dextran (1.0 × 10^−10^ M–1.0 × 10^−5^ M) were injected sequentially into the capacitive system. For each assay, the whole assay cycle including regeneration of the system and reconditioning was used. Each sample solution was injected three times. After injecting the samples into the capacitive sensor, an average of the last five readings before and after injection was calculated automatically. The calibration curves that show the capacitance change [ΔC (− pF·cm^−2^)] versus the logarithm of sugar concentration (M) are shown in Fig. 2. As seen from the figure, the capacitance change (∆C) increased linearly with the saccharide concentration in all cases. The response of the ∆C value and the studied glucose concentrations was ∆C = 1076,7×Conc + 8362.6 (R^2^ = 0.9792). The results show that the developed system is promising for sensitive glucose detection in a wide concentration range which is useful in applications where measurement of glucose levels is critical [40] such as cell cultures and microbial fermentation processes [49, 50].

For fructose, the response of the ∆C value and the fructose concentration was ∆C = 1799,3×conc + 13874 (R^2^ = 0.9773).

For dextran, a linear relationship between ∆C and concentration was obtained in the range of 1.0 × 10^−10^ M to 1.0 × 10^−5^ M with the regression equation of ∆C = 1511,7×conc + 15394 (R^2^ = 0.9248).

The limit of detection (LOD) values were determined as 8.0 × 10^−10^ M for glucose, 6.0 × 10^−10^ M for fructose and 13 × 10^−12^ M for dextran, based on the IUPAC guidelines [51].

A comparison of the LOD values with previous studies [15, 26, 27, 31, 52–56] is shown in Table 1. The LOD values obtained in this study are among the most sensitive values according to the table. When a comparison is made between the dynamic ranges, it can be clearly seen that the developed APBA–modified capacitive system can detect saccharides in a broader concentration range compared to the previous sensor systems.

**Table 1:** Comparison of analytical performances of different techniques based on phenylboronic acid used for saccharide detection

Sensing principle	Sensor preparation method	Linear range	Limit of detection (LOD)	Ref
Electrochemical impedance spectroscopy	Electropolymerizing 3-APBA on gold electrode surface	10^−9^ M-10^−2^ M for glucose,10^−10^ M-10^−2^ M for fructose, mannitol, sorbitol	N/D	[53]
pH-switchable bioelectrocatalytic sensor	Immobilization of glucose oxidase onto APBA moities which were covalently grafted onto mercaptobenzoic acid moities	0–30 × 10^−6^ M for glucose	348 × 10^−9^ M	[27]
Enzyme-free potentiometric sensor	Electrochemical preparation of poly (3-APBA-co-3-octylthiophene) organic electrode	5–50 × 10^−3^ M for glucose	5 × 10^−4^ M	[26]
Extended-gate type organic field effect transistor (OFET)	OFET functionalized by a phenylboronic acid monolayer	0–20 × 10^−3^ M for glucose, fructose and galactose	Higher than 5 × 10^−3^ M	[54]
Amperometric biosensor	Covalent immobilization of glucose oxidase onto poly (aniline boronic acid) modified electrode	0–20 × 10^−3^ M for glucose	24 × 10^−5^ M	[55]
Fluorescence titration	Fluorescence sensory membrane based on the design of an acrylic monomer with a phenylboronic acid residue conjugated with an aromatic imino group and a phenyl ring	4.76 × 10^−5^ M-1.01 × 10^−1^ M for glucose and fructose	3–4 × 10^−4^ M	[15]
Surface plasmon resonance	Glucose-modulated assembly of 5-amino-2-fluorophenylboronic acid–modified silver nanoparticles	0–20 × 10^−3^ M for glucose	89 × 10^−6^ M	[31]
UV_vis spectral measurement	Self-assembly of phenylboronic acid azoprobes on the surface of the polyamidoamine dendrimer in water	0–1 × 10^−3^ M for glucose, fructose and galactose	N/D	[56]
Fluorescence quenching	Nitrogen doped carbon quantum dots functionalized by phenylboronic acid	1–14 × 10^−3^ M for glucose	N/D	[52]
Capacitive biosensor	Preparation of APBA moieties onto tyramine-electropolymerized gold electrodes	10^−8^ M-10^−3^ M for glucose, 10^−8^ M-10^−2^ M for fructose,10^−10^ M-10^−5^ M for dextran	8.0 × 10^−10^ M for glucose, 6.0 × 10^−10^ M for fructose, 13 × 10^−12^ M for dextran	In this study

### Real-Time Glycoprotein Detection with APBA–Modified Capacitive Sensor

Since boronate selectively captures molecules with 1,2- and 1,3-cis-vicinal diol moieties which are mostly found in carbohydrates, it should be regarded as the first choice as an affinity ligand for cis-diol containing compounds [58, 59]. Therefore, boronate functionalized matrix that can capture glycol moieties through covalent binding with a 1,2-cis-diol of glycol-structure has been used as a general sorbent in affinity chromatography [60] for purification of glycoproteins and for the oriented immobilization of glycoproteins in cellulose supports [61].

In this study, human immunoglobulin G (IgG) was chosen as the model glycoprotein for quantifying glycoproteins with the APBA–modified capacitive system. The operating conditions were the same as the operating conditions for saccharide detection. Label-free IgG detection from aqueous IgG solutions was carried out with the APBA–modified electrode. IgG solutions containing different concentrations of IgG (1.0 × 10^−13^ M–1.0 × 10^−7^ M) were prepared in the running buffer and sequentially injected into the system. Triplicate measurements were performed for each concentration.

**Fig. 3:**
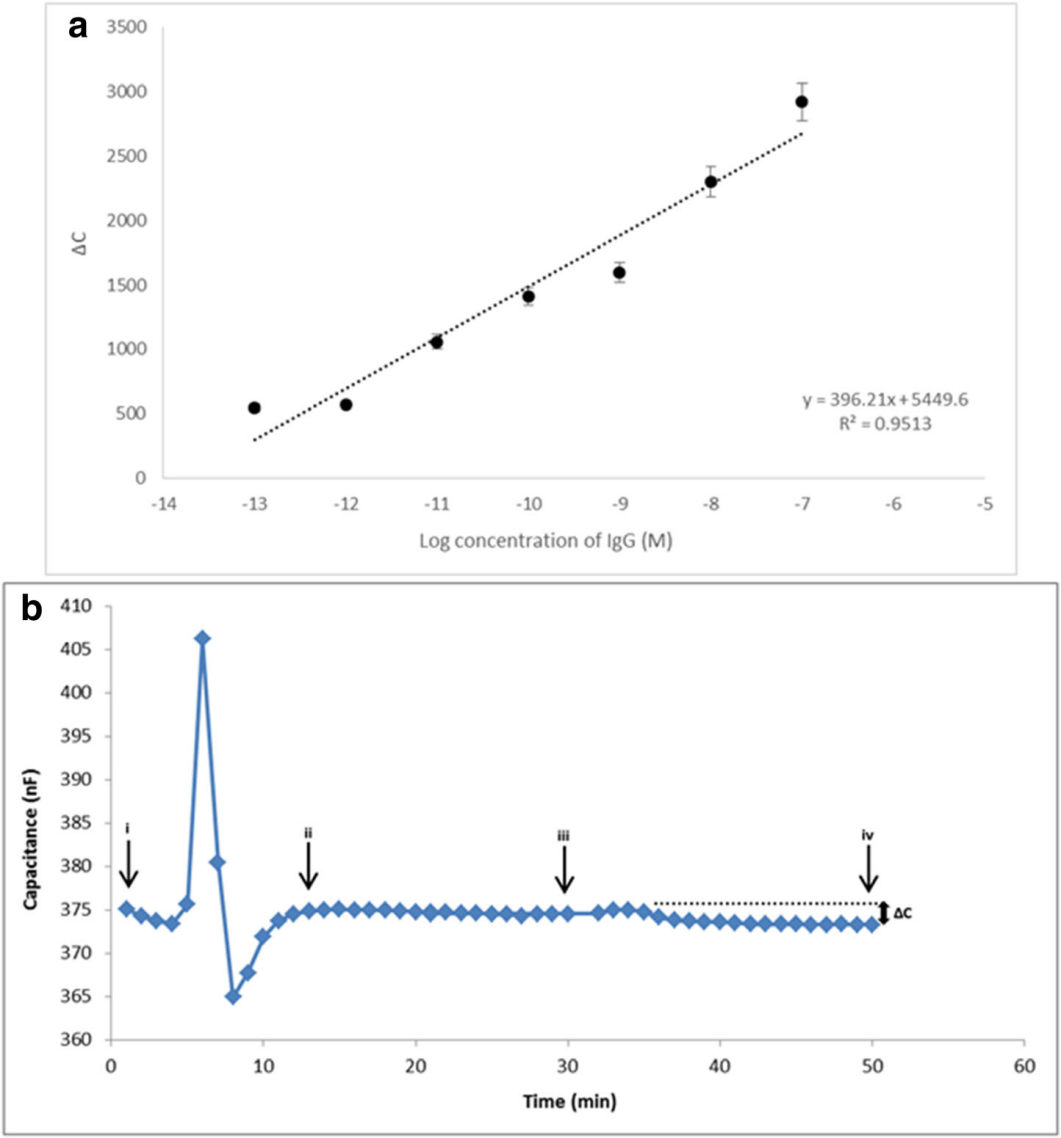
**A** Capacitance change vs. logarithm of IgG concentrations for APBA–modified electrode under optimum conditions. **B** Actual sensorgram showing the capacitance change of APBA–modified electrode after injection of IgG solution (1.0 × 10^−9^ M) under optimum conditions (i: before regeneration, ii: after regeneration, iii: before injection of IgG solution, iv: after injection and re-equilibration with the running buffer). The dotted line is an extension of the stable baseline before injection, and ∆C is the change in signal registered upon injection of a IgG containing sample

The sensorgram shown in Fig. 3B illustrates the decrease in capacitance signal that was registered upon binding of IgG to the electrode surface. The calibration curve for IgG (M) is shown in Fig. 3A. A linear relationship was obtained between the registered signal and the log concentration of IgG with regression equation of ∆C = 396,21×conc + 5449.6 (R^2^ = 0.9513). LOD value was calculated as 16 × 10^−15^ M based on IUPAC guidelines.

A comparison of the LOD values for glycoprotein detection obtained from previous phenylboronic acid–based recognition systems [15, 48, 57, 62–68] show that (Table 2), the LOD value and the dynamic range for IgG demonstrate that the developed capacitive system is promising to detect a glycoprotein in a very broad concentration range with high sensitivity. This comparison also shows the high sensitivity of capacitive sensors compared to other sensing platforms.

**Table 2:** Comparison of analytical performances of different techniques based on phenylboronic acid used for glycoprotein detection

Target analyte	Sensing principle	Sensor preparation method	Linear range	Limit of detection (LOD)	Ref
Dopamine	Electrochemical	Molecularly imprinted poly (acrylamidophenylboronic acid) film	5 × 10^−9^ M–2 × 10^−6^ M	20 × 10^−9^ M	[63]
Carcinoembryonic antigen (CEA)	Electrochemical	Self-assembling of a thiol-mixed monolayer comprised of 3-APBA with 11-mercaptoundecanoic acid and 11-mercapto-1-undecanol on gold	1.25–20 × 10^−15^ M	0.55 × 10^−15^ M	[48]
Dopamine	Impedimetric biosensor	Electropolymerization of 3-APBA on a preformed polyaniline layer	1 × 10^−10^ M–1 × 10^−5^ M	1 × 10^−10^ M	[57]
Ovalbumin	Electrochemical	Magnetic ferriferous oxide nanoparticles functionalized by phenylboronic acid	2.2 × 10^−8^ M–44 × 10^−8^ M	2.75 × 10^−9^ M	[67]
Prostate specific antigen (PSA)	Reusable amperometric immunosensor	Enzyme conjugated anti-PSA antibody reversible binding with a self-assembled phenylboronic acid monolayer on gold	6–45 × 10^−11^ M and 45–60 × 10^−11^ M	N/D	[64]
Dopamine	Imprinted electrochemical sensor	Molecular imprinting based sensor using pyrrole-phenylboronic acid as the electropolymerized monomer	5.0 × 10^−8^ M–1.0 × 10^−5^	3.3 × 10^−8^ M	[68]
Horse radish peroxidase (HRP)	Electrochemical	Self-assembly of 4-mercaptophenylboronic acid on dendritic gold nanoparticles	2.5 × 10^−9^ M–25 × 10^−6^ M	0.5 × 10^−9^ M	[66]
Adrenocorticotropic hormone (ACTH)	Electrochemical immunosensor	Use of APBA for the oriented immobilization of Anti-ACTH antibodies onto screen-printed carbon modified electrode surface	2.2 × 10^−16^ M–1.1 × 10^−13^ M	4.0 × 10^−17^ M	[65]
Dopamine	Fluorescence titration	Fluorescence sensory membrane based on the design of an acrylic monomer with a phenylboronic acid residue conjugated with an aromatic imino group and a phenyl ring	4.76 × 10^−5^ M–1.01 × 10^−1^ M	3–4 × 10^−4^ M	[15]
Pathogenic influenza A virus	Quartz crystal microbalance (QCM) and surface plasmon resonance (SPR)	Use of 4-aminophenyl boronic acid (4-APBA) as a ligand for binding of sialic acid (SA) via boronic acid–sugar interaction which then interacts with hemagglutinin protein on Influenza A virus	0.01 × 10^−3^ M–0.16 × 10^−3^ M	4.7 × 10^−8^ M for QCM and 1.28 × 10^−7^ M for SPR	[62]
Human immunoglobulin G (IgG)	Capacitive biosensor	Preparation of APBA moieties onto tyramine-electropolymerized gold electrodes	1.0 × 10^−13^ M–1.0 × 10^−7^ M	16 × 10^−15^ M	In this study

### Confirmation of Detection Performance of APBA–Modified Capacitive Sensor with Spectrophotometric Measurement

In order to confirm the detection performance of APBA–modified capacitive system, the amount of captured enzyme (HRP) quantified by the capacitive system was compared with spectrophotometric results by monitoring the enzymatic activity. In the first step, HRP was bound to the APBA–modified capacitive electrode and the amount of bound HRP was registered by using the change in capacitance. Then, this electrode with bound HRP was used to determine the enzymatic activity of HRP by using a spectrophotometric assay.

For this purpose, the HRP activity of the system was measured with a spectrophotometer at 650 nm after HRP was captured by the APBA–modified electrode, taken out and treated with the substrate (TMB+H_2_O_2_). One unit of enzyme was defined as the amount of enzyme catalyzing the conversion of 1 μmol of substrate (TMB) per minute at 25 °C and pH 7.0. By using this definition, the unit of HRP was measured as 5 mU/mL (0.0050 μmol·min^−1^) by spectrophotometer and it was measured as 5.4 mU/mL (0.0054 μmol·min^−1^) by the capacitive system. This result shows that the amount of captured enzyme detected by the APBA–modified capacitive sensor was correlative to the amount of captured enzyme spectrophotometrically measured.

## Conclusion

The work described herein is a model study to investigate the sensitive saccharide detection with aminophenylboronic acid (APBA) based capacitive sensor. High sensitivity, fast measurement, label-free detection, and low-cost are the main advantages of the system. Surface modification method is the main reason for the poor selectivity. However, selectivity can be improved by using multiboronic acids which possess multiple binding sites and thanks to the improvements in synthetic and supramolecular chemistry and materials science.

The developed system can recognize different types of saccharides with high affinities in a good linear relationship between registered signal and concentration of the target saccharide with limit of detection (LOD) values as 0.8 nM for glucose, 0.6 nM for fructose, and 13 pM for dextran. The system is suitable to operate in a broad concentration range with high sensitivity and can be used in cases where glucose is free from the interference of fructose or glucose in order to satisfy the selectivity requirement.

The developed capacitive system can also be used for very sensitive, label-free, and fast glycoprotein detection with a LOD value of 16 fM which is very promising for further applications where an ultrasensitive detection of a glycoprotein is required. Of special interest today is the level of glycosylation of produced proteins. By combining affinity separation based on the protein properties and a subsequent assay of level of glycosylation, it would be possible to get a sensitive and quick assay of the level of glycosylation.

## Electronic supplementary material

ESM 1 (DOCX 1334 kb)

## References

[1] Fukuda, M., & Hindsgaul, O. (1994). *Molecular glycobiology*. RL Press; Oxford University Press.

[2] El Rassi, Z. (1994). Carbohydrate analysis: high performance liquid chromatography and capillary electrophoresis, vol. 58. Elsevier.

[3] Soh N, Sonezaki M, Imato T (2003). Modification of a thin gold film with boronic acid membrane and its application to a saccharide sensor based on surface plasmon resonance. Electroanalysis.

[4] Cohen RM, Holmes YR, Chenier TC, Joiner CH (2003). Discordance between HbA1c and fructosamine: evidence for a glycosylation gap and its relation to diabetic nephropathy. Diabetes Care.

[5] Cohen, R.M., Haggerty, S., Herman, W.H. (2010). *HbA1c for the diagnosis of diabetes and prediabetes: is it time for a mid-course correction?* Oxford University Press. 10.1210/jc.2010-2352 PMC2999978 21131541

[6] Florkowski C (2013). HbA1c as a diagnostic test for diabetes mellitus–reviewing the evidence. The Clinical Biochemist Reviews.

[7] Wu X, Li Z, Chen XX, Fossey JS, James TD, Jiang YB (2013). Selective sensing of saccharides using simple boronic acids and their aggregates. Chemical Society Reviews.

[8] DiCesare N, Pinto MR, Schanze KS, Lakowicz JR (2002). Saccharide detection based on the amplified fluorescence quenching of a water-soluble poly (phenylene ethynylene) by a boronic acid functionalized benzyl viologen derivative. Langmuir.

[9] Egawa Y, Seki T, Takahashi S, Anzai JI (2011). Electrochemical and optical sugar sensors based on phenylboronic acid and its derivatives. Materials Science and Engineering: C.

[10] Granot E, Tel-Vered R, Lioubashevski O, Willner I (2008). Stereoselective and enantioselective electrochemical sensing of monosaccharides using imprinted boronic acid-functionalized polyphenol films. Advanced Functional Materials.

[11] Ivanov AE, Panahi HA, Kuzimenkova MV, Nilsson L, Bergenståhl B, Waqif HS, Jahanshahi M, Galaev IY, Mattiasson B (2006). Affinity adhesion of carbohydrate particles and yeast cells to boronate-containing polymer brushes grafted onto siliceous supports. Chemistry– A European Journal.

[12] Ivanov AE (2009). Boronate-containing polymer brushes: characterization, interaction with saccharides and mammalian cancer cells. Journal of Biomedical Materials Research Part A.

[13] Li Y, Larsson EL, Jungvid H, Galaev IY, Mattiasson B (2001). Shielding of protein–boronate interactions during boronate chromatography of neoglycoproteins. Journal of Chromatography A.

[14] Li Y, Pfüller U, Linné Larsson E, Jungvid H, Galaev IY, Mattiasson B (2001). Separation of mistletoe lectins based on the degree of glycosylation using boronate affinity chromatography. Journal of Chromatography A.

[15] Pablos JSL, Vallejos S, Ibeas S, Muñoz A, Serna F, García FC, García JM (2015). Acrylic polymers with pendant phenylboronic acid moieties as “turn-off” and “turn-on” fluorescence solid sensors for detection of dopamine, glucose, and fructose in water. ACS Macro Letters.

[16] Egawa Y, Miki R, Seki T (2014). Colorimetric sugar sensing using boronic acid-substituted azobenzenes. Materials.

[17] Hargrove AE, Reyes RN, Riddington I, Anslyn EV, Sessler JL (2010). Boronic acid porphyrin receptor for ginsenoside sensing. Organic Letters.

[18] James TD, Linnane P, Shinkai S (1996). Fluorescent saccharide receptors: a sweet solution to the design, assembly and evaluation of boronic acid derived PET sensors. Chemical Communications.

[19] Ozawa R (2008). Effect of cyclodextrins on saccharide sensing function of a fluorescent phenylboronic acid in water. Analytical Sciences.

[20] Suri JT, Cordes DB, Cappuccio FE, Wessling RA, Singaram B (2003). Continuous glucose sensing with a fluorescent thin-film hydrogel. Angewandte Chemie International Edition.

[21] Yoon J, Czarnik AW (1992). Fluorescent chemosensors of carbohydrates. A means of chemically communicating the binding of polyols in water based on chelation-enhanced quenching. Journal of the American Chemical Society.

[22] Gabai R, Sallacan N, Chegel V, Bourenko T, Katz E, Willner I (2001). Characterization of the swelling of acrylamidophenylboronic acid-acrylamide hydrogels upon interaction with glucose by faradaic impedance spectroscopy, chronopotentiometry, quartz-crystal microbalance (QCM), and surface plasmon resonance (SPR) experiments. The Journal of Physical Chemistry B.

[23] Lee M, Kim TI, Kim KH, Kim JH, Choi MS, Choi HJ, Koh K (2002). Formation of a self-assembled phenylboronic acid monolayer and its application toward developing a surface plasmon resonance-based monosaccharide sensor. Analytical Biochemistry.

[24] Stephenson-Brown A, Wang HC, Iqbal P, Preece JA, Long Y, Fossey JS, James TD, Mendes PM (2013). Glucose selective surface plasmon resonance-based bis-boronic acid sensor. Analyst.

[25] Aytaç S, Kuralay F, Boyacı İH, Unaleroglu C (2011). A novel polypyrrole–phenylboronic acid based electrochemical saccharide sensor. Sensors and Actuators B: Chemical.

[26] Çiftçi H, Tamer U, Teker MŞ, Pekmez NÖ (2013). An enzyme free potentiometric detection of glucose based on a conducting polymer poly (3-aminophenyl boronic acid-co-3-octylthiophene). Electrochimica Acta.

[27] Gao P, Wang Z, Yang L, Ma T, Yang L, Guo Q, Huang S (2015). A glucose-responsive pH-switchable bioelectrocatalytic sensor based on phenylboronic acid-diol specificity. Electrochimica Acta.

[28] Ates M, Sarac AS (2009). Conducting polymer coated carbon surfaces and biosensor applications. Progress in Organic Coatings.

[29] Mello LD, Kubota LT (2002). Review of the use of biosensors as analytical tools in the food and drink industries. Food Chemistry.

[30] Ramanavičius A, Ramanavičienė A, Malinauskas A (2006). Electrochemical sensors based on conducting polymer—polypyrrole. Electrochimica Acta.

[31] Cao K, Jiang X, Yan S, Zhang L, Wu W (2014). Phenylboronic acid modified silver nanoparticles for colorimetric dynamic analysis of glucose. Biosensors and Bioelectronics.

[32] Irshad M, Mujahid A, Afzal A, Bajwa SZ, Hussain T, Zaman WU, Latif U, Athar MM (2018). A miniaturized electronic sensor for instant monitoring of ethanol in gasohol fuel blends. RSC Advances.

[33] Ertürk G, Hedström M, Tümer MA, Denizli A, Mattiasson B (2015). Real-time prostate-specific antigen detection with prostate-specific antigen imprinted capacitive biosensors. Analytica Chimica Acta.

[34] Ertürk G, Hedström M, Mattiasson B (2016). A sensitive and real-time assay of trypsin by using molecular imprinting-based capacitive biosensor. Biosensors and Bioelectronics.

[35] Hedström M, Galaev IY, Mattiasson B (2005). Continuous measurements of a binding reaction using a capacitive biosensor. Biosensors and Bioelectronics.

[36] Mattiasson B, Teeparuksapun K, Hedström M (2010). Immunochemical binding assays for detection and quantification of trace impurities in biotechnological production. Trends in Biotechnology.

[37] Ertürk G, Lood R (2018). Bacteriophages as biorecognition elements in capacitive biosensors: phage and host bacteria detection. Sensors and Actuators B: Chemical.

[38] Ertürk G, Berillo D, Hedström M, Mattiasson B (2014). Microcontact-BSA imprinted capacitive biosensor for real-time, sensitive and selective detection of BSA. Biotechnology Reports.

[39] De Guzman JM, Soper SA, McCarley RL (2010). Assessment of glycoprotein interactions with 4-[(2-aminoethyl) carbamoyl] phenylboronic acid surfaces using surface plasmon resonance spectroscopy. Analytical Chemistry.

[40] Labib M (2010). A novel competitive capacitive glucose biosensor based on concanavalin A-labeled nanogold colloids assembled on a polytyramine-modified gold electrode. Analytica chimica acta.

[41] Lebogang L, Mattiasson B, Hedström M (2014). Capacitive sensing of microcystin variants of microcystis aeruginosa using a gold immunoelectrode modified with antibodies*,* gold nanoparticles and polytyramine. Microchimica Acta.

[42] Hedström M, Mattiasson B (2016). Bioimprinting as a tool for the detection of aflatoxin B1 using a capacitive biosensor. Biotechnology Reports.

[43] Erlandsson D, Teeparuksapun K, Mattiasson B, Hedström M (2014). Automated flow-injection immunosensor based on current pulse capacitive measurements. Sensors and Actuators B: Chemical.

[44] John Goka A, Farthing MJ (1987). The use of 3, 3′, 5, 5′-tetramethylbenzidine as a peroxidase substrate in microplate enzyme-linked immunosorbent assay. Journal of Immunoassay.

[45] Teeparuksapun K, Hedström M, Wong EY, Tang S, Hewlett IK, Mattiasson B (2010). Ultrasensitive detection of HIV-1 p24 antigen using nanofunctionalized surfaces in a capacitive immunosensor. Analytical Chemistry.

[46] Teeparuksapun K, Hedström M, Kanatharana P, Thavarungkul P, Mattiasson B (2012). Capacitive immunosensor for the detection of host cell proteins. Journal of Biotechnology.

[47] Berggren C, Bjarnason B, Johansson G (2001). Capacitive biosensors. Electroanalysis: An International Journal Devoted to Fundamental and Practical Aspects of Electroanalysis.

[48] Zhang X, Wu Y, Tu Y, Liu S (2008). A reusable electrochemical immunosensor for carcinoembryonic antigen via molecular recognition of glycoprotein antibody by phenylboronic acid self-assembly layer on gold. Analyst.

[49] Choy V, Patel N, Thibault J (2007). Blood glucose monitor: an alternative off-line method to measure glucose concentration during fermentations with Trichoderma reesei. Biotechnology Letters.

[50] Liu Y-C, Wang FS, Lee WC (2001). On-line monitoring and controlling system for fermentation processes. Biochemical Engineering Journal.

[51] McNaught AD, Wilkinson A (1997). Compendium of chemical terminology: IUPAC.

[52] Jiang G, Jiang T, Li X, Wei Z, du X, Wang X (2014). Boronic acid functionalized N-doped carbon quantum dots as fluorescent probe for selective and sensitive glucose determination. Materials Research Express.

[53] Ma Y, Yang X (2005). One saccharide sensor based on the complex of the boronic acid and the monosaccharide using electrochemical impedance spectroscopy. Journal of Electroanalytical Chemistry.

[54] Minami T, Minamiki T, Hashima Y, Yokoyama D, Sekine T, Fukuda K, Kumaki D, Tokito S (2014). An extended-gate type organic field effect transistor functionalised by phenylboronic acid for saccharide detection in water. Chemical Communications.

[55] Şenel M, Nergiz C, Dervisevic M, Çevik E (2013). Development of amperometric glucose biosensor based on reconstitution of glucose oxidase on polymeric 3-aminophenyl boronic acid monolayer. Electroanalysis.

[56] Tsuchido Y, Sakai Y, Aimu K, Hashimoto T, Akiyoshi K, Hayashita T (2015). The design of phenylboronic acid azoprobe–polyamidoamine dendrimer complexes as supramolecular sensors for saccharide recognition in water. New Journal of Chemistry.

[57] Plesu N, Kellenberger A, Taranu I, Taranu BO, Popa I (2013). Impedimetric detection of dopamine on poly (3-aminophenylboronic acid) modified skeleton nickel electrodes. Reactive and Functional Polymers.

[58] James TD, Sandanayake K, Shinkai S (1996). Saccharide sensing with molecular receptors based on boronic acid. Angewandte Chemie International Edition in English.

[59] Liu S, Miller B, Chen A (2005). Phenylboronic acid self-assembled layer on glassy carbon electrode for recognition of glycoprotein peroxidase. Electrochemistry Communications.

[60] Soundararajan S, Badawi M, Kohlrust CM, Hageman JH (1989). Boronic acids for affinity chromatography: spectral methods for determinations of ionization and diol-binding constants. Analytical Biochemistry.

[61] Abad JM (2002). Immobilization of peroxidase glycoprotein on gold electrodes modified with mixed epoxy-boronic acid monolayers. Journal of the American Chemical Society.

[62] Diltemiz SE (2013). 4-Aminophenyl boronic acid modified gold platforms for influenza diagnosis. Materials Science and Engineering: C.

[63] Hong S, Lee LYS, So MH, Wong KY (2013). A dopamine electrochemical sensor based on molecularly imprinted poly (acrylamidophenylboronic acid) film. Electroanalysis.

[64] Liu S, Zhang X, Wu Y, Tu Y, He L (2008). Prostate-specific antigen detection by using a reusable amperometric immunosensor based on reversible binding and leasing of HRP-anti-PSA from phenylboronic acid modified electrode. Clinica Chimica Acta.

[65] Moreno-Guzmán M, Ojeda I, Villalonga R, González-Cortés A, Yáñez-Sedeño P, Pingarrón JM (2012). Ultrasensitive detection of adrenocorticotropin hormone (ACTH) using disposable phenylboronic-modified electrochemical immunosensors. Biosensors and Bioelectronics.

[66] Shen D, Liu Y, Fang Y, Li P, Yang Z (2015). A sensor for glycoproteins based on dendritic gold nanoparticles electrodeposited on a gold electrode and modified with a phenylboronic acid. Journal of Solid State Electrochemistry.

[67] Sun S, Xiong L, Li Y, He X (2015). Phenylboronic acid modified magnetic nanoparticles for the electrochemical determination of glycoproteins. Analytical Letters.

[68] Zhong M, Teng Y, Pang S, Yan L, Kan X (2015). Pyrrole–phenylboronic acid: A novel monomer for dopamine recognition and detection based on imprinted electrochemical sensor. Biosensors and Bioelectronics.

