# Supplementary material for: Capacitive Saccharide Sensor Based on Immobilized Phenylboronic Acid with Diol Specificity
Source: Appl Biochem Biotechnol. 2018 Oct 28;188(1):124–37. doi: 10.1007/s12010-018-2911-3 (PMC6509085; doi:10.1007/s12010-018-2911-3)
Supplement: Supplementary file 1 — (DOCX 1334 kb) [file 12010_2018_2911_MOESM1_ESM.docx]

**SUPPLEMENTARY INFORMATION**

**Capacitive Saccharide Sensor Based on Immobilized Phenylboronic Acid with Diol Specificity**

**Gizem Ertürk Bergdahl^a,b,c*^, Martin Hedström^a,b^, Bo Mattiasson^a,b^**

**^a^CapSenze Biosystems AB, Scheelevägen 22, 22363 Lund, Sweden**

**^b^Department of Biotechnology, Kemicentum, Lund University, Sölvegatan 39A, 22100 Lund, Sweden**

**^c^Department of Clinical Sciences, Lund University, Tornavägen 10, 22184, Lund, Sweden**

**Surface characterization of APBA-modified electrodes with CV, AFM and SEM**


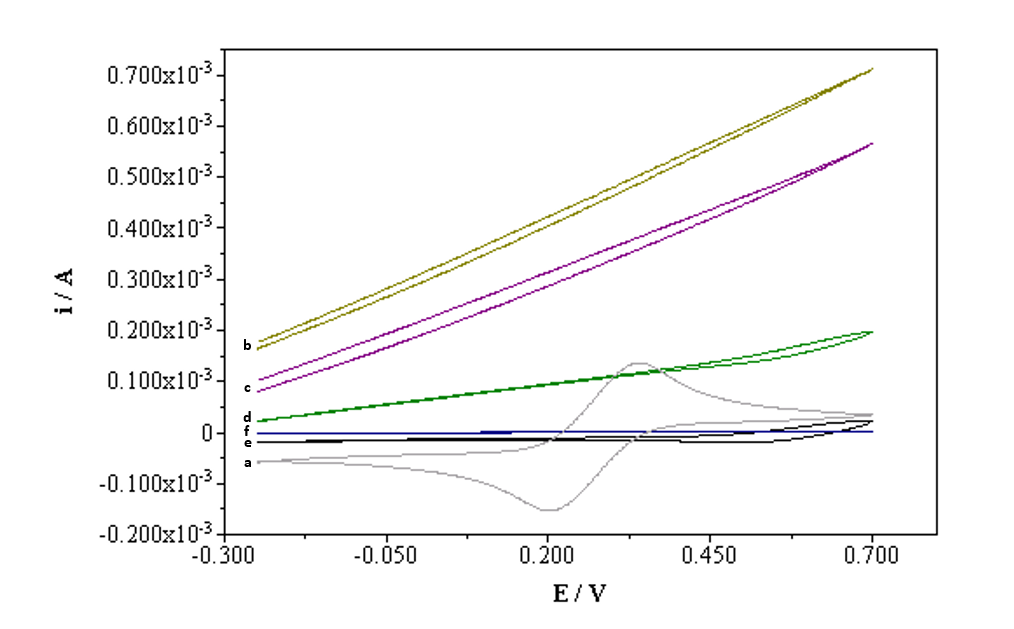
The electrochemical characterization of the modified surface is shown in Figure SI.1.

**Figure SI.1.** Characterization of electrodes with cyclic voltammogram: bare gold electrode (a-grey), gold electrode after electro-polymerization of tyramine (b-yellow), gold electrode after carboxymethyl cellulose (CMC) activation (c-purple), gold electrode after carboxylic group of CMC was activated with EDC/NHS (d-green), gold electrode after NHS-activated groups were exposed and bonded to 3-APBA (e-black), gold electrode after treatment with 1-dodecanethiol (f-blue).

As seen from the figure, the clean, bare gold surface showed large redox peaks (a-grey). The amplitude decreased after tyramine electro-polymerization (b-yellow) and carboxymethyl cellulose (CMC) activation (c-purple) on the gold surface. Further reduction of the redox peaks was observed when the carboxylic group of CMC was activated with EDC/NHS (d-green). As seen from the figure, the peaks almost disappeared when the activated groups were exposed and reacted with 3-APBA (e-black). Finally, treatment with 1-dodecanethiol resulted in a highly blocked interface (f-blue). The long aliphatic chain of 1-dodecanethiol is used to obtain a completely insulated electrode surface. The electrochemical results indicate that a complete, tight coverage of the APBA-modified electrode was provided and the electrode could be used for analysis.

**D**

**C**

**B**

**A**


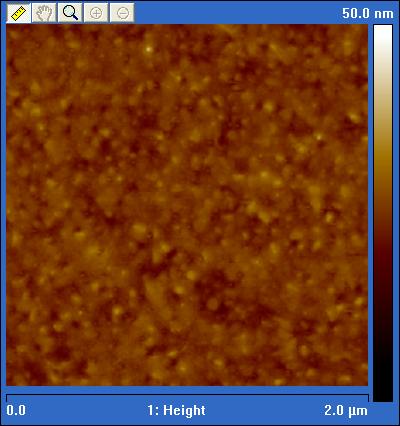

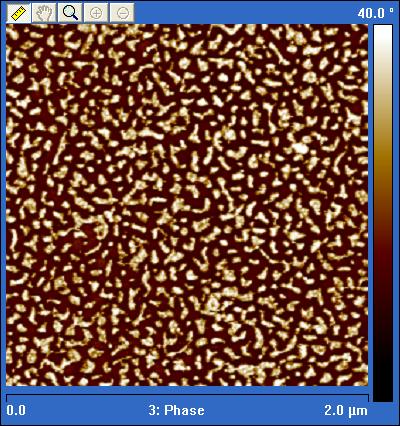

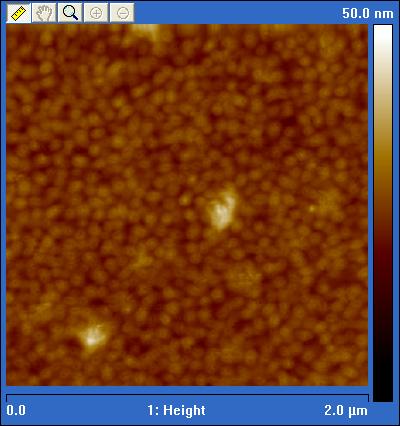

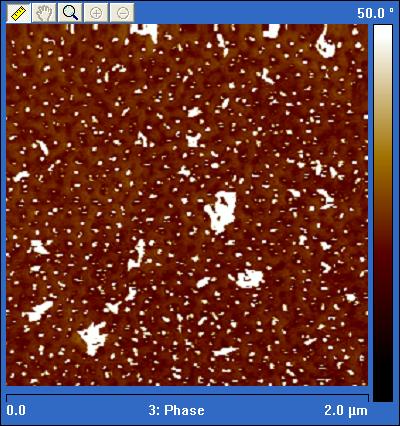


**Figure SI.2.** AFM images of (A-B) bare gold electrode, (C-D) APBA-modified electrode

Figure SI.3 shows the SEM images of APBA-modified electrode in different magnifications. The rough surface can be clearly seen in the images and it indicates the successful modification of the surface with CMC + APBA.


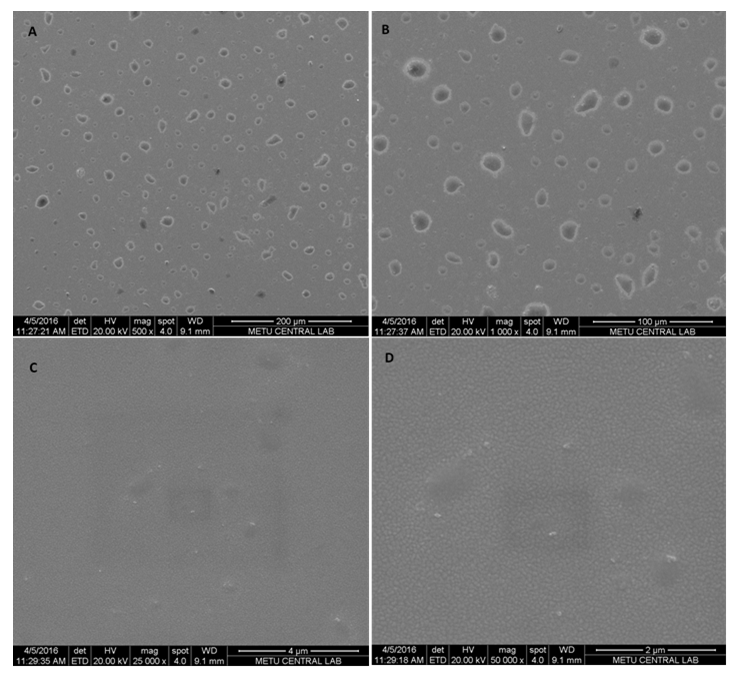


**Figure SI.3.** SEM images of APBA-modified electrodes in different magnifications (A-500X), (B-1000X), (C-25000X), (D-50000X).

The SEM images of the bare gold surface in different magnifications are shown in Figure SI.4.


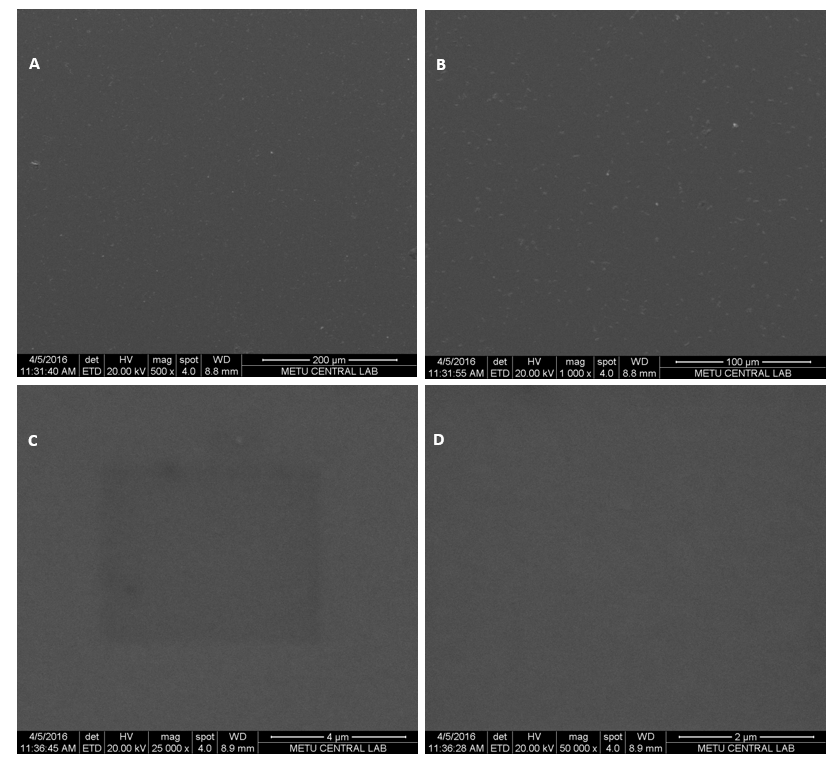


**Figure SI.4.** SEM images of bare gold electrode in different magnifications (A: 500X), (B: 1000X), (C: 25000X), (D: 50000X)
